# Role of poly-ADP-ribose polymerase inhibitors after brain progression in platinum-sensitive ovarian cancer: a case report and review of the literature

**DOI:** 10.3389/fonc.2024.1423992

**Published:** 2024-08-02

**Authors:** Gonzalo Lendinez-Sanchez, Tamara Diaz-Redondo, Marcos Iglesias-Campos, Lucía Garrido-Almazán, Emilio Alba-Conejo, Antonio Rueda-Dominguez, Alfonso Sanchez-Muñoz

**Affiliations:** ^1^ Medical Oncology, Intercenter Unit, Regional and Virgen de la Victoria University Hospitals, Instituto de Investigación Biomédica de Málaga (IBIMA), Malaga, Malaga, Spain; ^2^ Faculty of Medicine, University of Malaga, Malaga, Spain

**Keywords:** PARPi, olaparib, ovarian cancer, brain metastases, BRCA, case report, maintenance

## Abstract

**Introduction:**

The incidence of brain metastases in ovarian cancer is quite rare, being approximately 1%–2%. According to retrospective studies, patients with BRCA 1/2 mutations present a higher risk. The trimodal approach based on surgery, radiotherapy, and chemotherapy presents better outcomes, but the prognosis remains poor with overall survival since the brain progression is around 1 year. Poly-ADP-ribose polymerase inhibitors (PARPi) have provided a new alternative for the management of advanced ovarian cancer. The SOLO2, NOVA, and ARIEL3 clinical trials do not refer data on patients with brain metastases, and the published evidence for PARPi in this setting comes only from case reports and retrospective studies.

**Case report:**

We present the case of a 54-year-old woman with stage IV ovarian high-grade serous papillary carcinoma who, after 37 months of treatment with olaparib, presented a single brain lesion. After radical treatment with surgery and adjuvant whole-brain radiotherapy, she resumed olaparib with no evidence of disease during 15 months. After a second single brain relapse treated with stereotactic radiosurgery, the patient continued olaparib beyond the brain progression with no evidence of extracranial disease. Despite that there were no changes in size or number of brain lesions, the neurological situation progressively worsened and the patient died 8 months after the second progression.

**Discussion:**

The higher incidence of brain metastases of ovarian cancer points out a possible tropism for the CNS in BRCA-mutated patients. In preclinical studies, PARPi has shown to cross the blood–brain barrier, with possible antitumor activity in the central nervous system (CNS) while maintaining control of extracranial disease. The best survival data are obtained with a trimodal approach, and adding a PARPi could improve the survival outcomes in the context of platinum-sensitivity disease. Targeted therapies combined with local treatments are also used in other malignancies, suggesting potential effectiveness due to tumor heterogeneity. PARPi before brain metastasis may delay its diagnosis, and using iPARP after brain metastases could improve the outcome of this population.

**Conclusion:**

The role that PARPi may have in the treatment of brain metastases of ovarian cancer requires more studies. In the context of radical treatment of brain metastasis (surgery and/or RT), with no evidence of extracranial disease, maintaining treatment with PARPi beyond the brain progression should be considered.

## 1 Introduction

Managing brain metastases (BM) in cancer patients is challenging. The incidence of BM in ovarian cancer (OC) is quite low, being approximately 1.34% (0.49%–6.1%) (1). Despite the variety of existing treatments (surgery, radiotherapy, and chemotherapy), the survival remains very poor with overall survival (OS) around 8–12 months since the diagnosis (2) and there is no established guideline or protocol for their management.

In 15% of cases of high-grade serous ovarian cancer, there is a germline mutation of the BRCA1/2 gene, in 6% of cases a somatic mutation of the BRCA1/2 gene, and in 20% of cases a mutation of other genes involved in homologous recombination, for example, mutations and/or epigenetic silencing of the genes TR, ATM, RAD51/54, CHK1/2, NBS1, PALB2, and PTEN and which equally determine a profile defined as “BRCA-ness” (3, 4). These mutations cause the cell to lose the ability to repair DNA damage caused by external insults, specifically those to the double helix, resulting in a condition defined as homologous recombination deficiency (HRD). This occurrence favors tumor initiation, growth, and evolution. With the acquisition of such molecular, genetic, and biological knowledge, in the last decade, a class of drugs has been identified (4).

Poly-ADP-ribose polymerase inhibitors (PARPi) offer a new option for managing advanced ovarian cancer (OC) in patients with homologous recombination deficiency (HRD) (5–7). However, there is insufficient evidence regarding their effectiveness in ovarian cancer with brain metastases (BM).We present the case of a 54-year-old woman diagnosed with high-grade papillary serous carcinoma of the ovary with a pathogenic germline mutation in BRCA2. During maintenance treatment with olaparib, she developed a single brain lesion consistent with metastasis. After radical surgical excision, she continued olaparib and achieved a radiological complete response (CR) for 15 months. Due to the difficulty of conducting randomized prospective studies in patients with these characteristics, we report our experience and review the current literature based on case reports and retrospective series.

## 2 Case description

A 54-year-old woman with no significant medical or surgical history was diagnosed with ovarian neoplasia after postmenopausal metrorrhagia. She underwent suboptimal cytoreductive surgery for high-grade serous carcinoma involving both ovaries, the uterus, and multiple peritoneal implants. Malignant cells were found in the ascitic fluid, and thoracic tomography showed pleural thickening with associated pleural effusion, with cytology positive for papillary adenocarcinoma.

The patient’s ovarian cancer was initially staged as IVA stage, with a postsurgical CA125 serum level at 646 mg/dl. She received seven cycles of chemotherapy with paclitaxel 175 mg/m^2^ and carboplatin (AUC 6)–bevacizumab 7.5 mg/kg, achieving a partial response (PR) and normalization of CA125 serum level. Subsequently, she continued bevacizumab for 16 cycles, reaching radiological complete response (CR). Four months after stopping bevacizumab, she experienced peritoneal progression and was switched to cisplatin–paclitaxel, receiving four cycles achieving radiological CR and normalization of tumor markers. A BRCA2 germline mutation (c.2636_2637delCT (p.Ser879Terfs)) was identified, and she started maintenance with olaparib 400 mg/12 h. In August 2021, 37 months from olaparib treatment, she had tonic-clonic seizures, with a 5 cm × 4 cm right frontal brain lesion detected. A multidisciplinary committee recommended complete excision confirming metastasis of high-grade serous papillary carcinoma positive for CK7+, WT1+, and RE+; CK20−. She received adjuvant whole-brain radiotherapy (WBRT) 30 Gy delivered in 10 fractions of 3 Gy each. In January 2022 with no systemic disease, olaparib was restarted at 250 mg/12 h. In April 2023, the patient reported gait instability and headache. A brain local relapse of 13 × 5 mm was detected in the MRI, and she was treated with stereotactic radiosurgery (SRS). After SRS, she experienced slight clinical improvement and continued olaparib. The following brain MRI showed persistence of local recurrence and signs of post-radiotherapy encephalopathy. Despite no changes in size or number of brain lesions, the patient’s neurological condition progressively worsened, leading to inability to walk, loss of sphincters, disorientation, and stupor. The patient died in December 2023.

**Figure 1:**
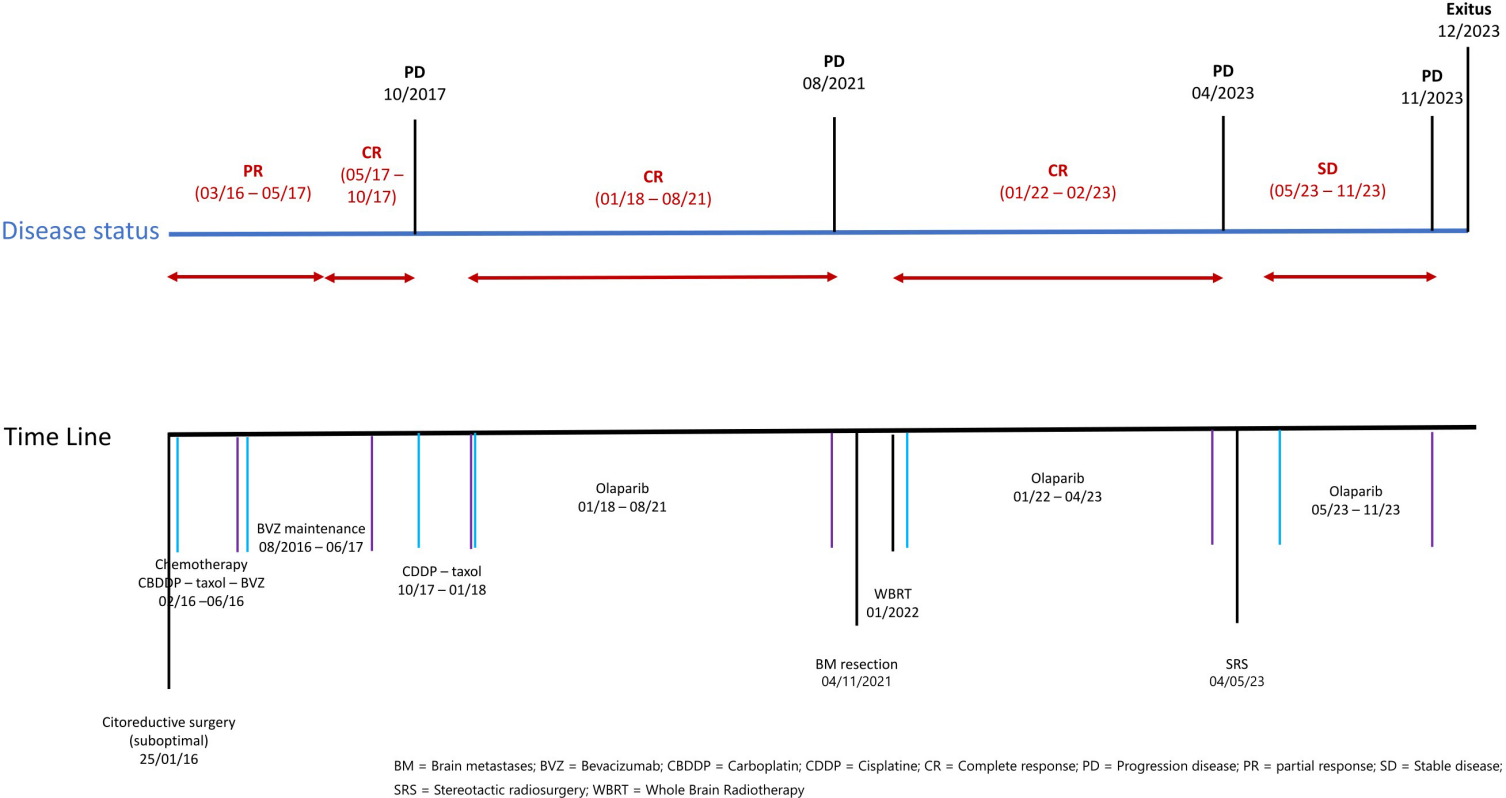
Time line of treatments and disease situation.

The treatment and progression periods are summarized in **Figure 1**.

## 3 Discussion

We present a case in which after a solitary brain metastasis successfully treated by surgery and radiotherapy, maintenance with olaparib was resumed given the absence of extracranial disease and remaining without progression for 15 months.

The incidence of brain metastases in ovarian cancer is approximately 1.34% (0.49%–6.1%) (1, 8–10). In a retrospective study carried out in Israel (with 58.6% of patients of Ashkenazi Jewish ethnicity), the rate of BM in patients with BRCAm was higher than the rate of BM in patients with BRCAwt (5.1% vs. 2.1%, p = 0.013), with an OR of 2.6 (9). In another retrospective study, the hazard ratio (HR) for developing brain metastases in patients carrying BRCA mutations was 3.84 (95% CI: 1.60–9.22, p < 0.001) with no difference in OS between BRCAm and BRCAwt (11, 12). These data support a higher incidence of BM from OC, pointing out a possible tropism for the CNS in BRCAm patients.

In a retrospective study of 174 women with BM from OC, the best survival data are obtained with a trimodal approach (radiation therapy, surgery, and chemotherapy) whereas monotherapy treatment is associated with poorer survival (HR: 2.57, 95% CI: 1.64–3.86) (2). Despite multidisciplinary treatment, the prognosis remains poor with median OS around 1 year since brain progression (1, 12–14). Regarding systemic treatment, platinum derivatives remain the best therapeutic option in ovarian epithelial neoplasms and are capable of crossing the blood–brain barrier (BBB), occasionally achieving adequate control of the disease (2, 12).

PARPi induce the formation of double-stranded DNA breaks by trapping PARP1 and blocking the single-stranded DNA break repair pathway. Tumor cells deficient in HRD pathway eventually die due to the inability to accurately repair DNA double-strand breaks, known as synthetic lethality (4, 15, 16).

Identifying the BRCAness/HRD phenotype is clinically important to optimize the benefit of PARPi (4). The execution of the BRCA test is recommended to identify patients who derive most benefit from PARPi; in the context of the maintenance setting after first-line chemotherapy, it is also recommended to perform the HRD test to establish the extent of the benefit from PARPi in BRCAwt and platinum-sensitive patients (4).

The SOLO2 (olaparib), NOVA (niraparib), and ARIEL3 (rucaparib) clinical trials demonstrated an increase in progression-free survival (PFS) in patients with BRCA mutations compared with placebo: 19.1 vs. 5.5 (HR: 0.30); 21 vs. 5.5 (HR: 0.27); and 16.6 vs. 5.4 (HR: 0.23) when PARPi agents were administered as maintenance therapy in platinum responders (5–7). In addition, niraparib and rucaparib demonstrated increased PFS in the HRD subgroup and in the all-patient subgroup (5, 7).

A meta-analysis carried out in patients with platinum-sensitive recurrent OC confirmed the efficacy of PARPi in the recurrent OC BRCA-mutated population and in the HRD population (17). The current challenge is to investigate the potentiality of combined PARPi with other therapeutic agents in order to enhance efficacy and avoid resistance (4). Despite the presence of a highly immunosuppressive tumor microenvironment that causes poor recognition of tumor cells by the immune system, it is shown that BRCA-mutated and HRD ovarian cancers express higher levels of neoantigens because of defect in DNA repair mechanisms. Moreover, PARP inhibitors are suggested to upregulate PD-L1 expression and stimulate interferon-mediated immune response having therefore a synergic action in immune stimulation (4).

However, these clinical trials and meta-analysis do not make reference to data on patients with brain metastases. The PARPi data published about this population come mainly from clinical cases (8, 10, 15, 18–23) whose main characteristics are presented in **Table 1**. PFS is defined as time from treatment with PARPi until clinical/radiological progression, even if they continued PARPi beyond progression.

**Table 1:** Clinical cases of epithelial ovarian cancer with CNS disease treated with iPARPs published in PubMed.

Reference	Histology	Mutation	CNS disease	Management	iPARP	PFS	Best response
Zhang Z_6_	HGSC	Wild type	Single	Surgery	Niraparib	>29 m	CR
Kasherman_8_	HGSC	BRCA1g	Multiple	SRS+Cht	Olaparib	10 m	SD
Tao M_13_	HGSC	BRCA2s ATMg	Multiple	WBRT + Cht	Niraparib	>15 m	PR
Gray S_15_	SC	BRCA1g	Multiple	WBRT + Cht	Niraparib	>17 m	SD
Gallego A_16_	HGSC	BRCA1g	Multiple	WBRT + Cht	Olaparib	>42 m	PR
Bangham M_17_	ADC	BRCA2	Lepto-meningeal	Surgery + Cht + RT	Olaparib	12 m	PR
Sakamoto I_18_	PPC	BRCA1	Multiple	WBRT + Cht	Olaparib	>18 m	PR
Morales F_19_	HGSC	BRCA1	Multiple	WBRT + Cht	Olaparib	9 m	SD
Cabitza E_20_	HGSC	Wild Type	Single	Surgery + Cht	Niraparib	>13 m	CR
**Lendínez G**	**HGSC**	**BRCA2g**	**Single**	**Surgery+WBRT**	**Olaparib**	**15 m**	**CR**

ADC, adenocarcinoma; ATM, ataxia telangiectasia mutated; BRCA, breast cancer gene; Cht, chemotherapy; CNS, central nervous system; CR, complete response; g, germline; HGSC, high-grade serous carcinoma; iPARP, inhibitors of Poly ADP ribose polymerase; PFS, progression-free survival (since iPARP); PPC, primary peritoneal cancer; PR, partial response; s, somatic; SC, serous cystoadenocarcinoma; SD, stable disease; SRS, stereotactic radiosurgery; WBRT, whole-brain radiotherapy.

Except for the case presented by Morales (22), all these cases started PARPi in a platinum-sensitive situation. A platinum-sensitive setting has been reported to be a good prognostic factor in patients with BM from OC with an HR of 0.23 in comparation in a platinum-refractory setting (24).

This could be one of the reasons why the duration of the response has been longer in the cases shown in **Table 1**, compared with the OS data reported around 8–12 months reported in the literature (1, 2, 24). Recently, a study conducted in eight centers in the UK explored the role of PARPi in the management of BM from OC. A total of 29 patients were treated with PARPi, and for the patients whose treatments included PARPi therapy, the median OS was statistically better whether they had additional chemotherapy alone or triplet therapy, achieving the highest OS with chemotherapy + PARPi + other (RT and/or surgery) (14). The authors also pointed out that this better prognosis could be related to a first platinum-sensitive relapse (14).

Another retrospective study of 111 patients with ovarian cancer and brain metastasis analyzed BRCA status, surgical approach, and PARPi treatment. They found that receiving PARPi before brain metastasis delays its diagnosis but negatively affects post-brain metastasis survival in the BRCAm population. However, using PARPi after brain metastasis significantly improves survival expectations. In this cohort, five patients developed brain metastasis during maintenance treatment, and three of them continued PARPi after the diagnosis (25).

Our patient presented BM during maintenance treatment with olaparib. The singularity of the case allowed radical treatment of this lesion while remaining in CR at the extracranial level. In the context of radical treatment of brain metastasis (surgery and/or RT), with no evidence of extracranial disease, a reasonable option could be to maintain treatment with PARPi. In this setting, the role of PARPi has not been described, however, using targeted therapies beyond intracranial progression, which can be seen in other malignancies (10). As discussed in the case presented by Kasherman et al. where olaparib was maintained after cerebral progression, consideration should be given to continuing poly ADP-ribose polymerase inhibitors beyond localized disease control in ovarian high-grade serous carcinoma extracranial oligometastatic progression, given that progression in this context likely occurs within the context of clonal heterogeneity (10). Local treatment such as stereotactic body radiotherapy (SBRT) achieves good local control. In a retrospective study with data of 449 lesions from 261 patients with oligo-recurrent, persistent OC for which salvage surgery or other local therapies were not feasible due to any relative contra-indication to further systemic therapy, SBRT or SRS achieved a rate of 65.2% CR of irradiated lesions (26). After local disease control, several large poly ADP-ribose polymerase inhibitor studies continued treatment beyond progression (10, 27). In our case, after PFS of 15 months, the second intracranial relapse was managed with SRS, continuing olaparib with no evidence of extracranial disease until the patient’s death.

Regarding the differences between the PARPi, niraparib has shown to be able to cross the BBB in animal models (12), being superior to other PARPi. According to a preclinical study in rodents, concentration–time profiles of niraparib in the brain were similar to those in plasma, with mean brain-to-plasma concentration ratios of 0.85–0.99 indicating effective brain penetration in rodents. Niraparib showed significant antitumor activity in both subcutaneous and intracranial xenograft models, with tumor growth inhibition values up to 83%, supporting its potential clinical use against BRCA-mutant tumors metastasized to the brain (28).

Despite the fact that the pharmacodynamic data of olaparib showed little activity at the CNS (29), our case and other similar cases described in the bibliography show a clinical benefit of olaparib in patients with BM from OC, especially in patients with a BRCA 1/2 mutation (14, 19–22); however, the current evidence base in cases and retrospectives studies is not enough to confirm this statement.

## 4 Conclusion

- Brain metastases from OC are rare, representing a clinical challenge whose management requires a multidisciplinary approach. The emergence of PARPi has provided clinicians with new therapeutic options to enhance the prognosis of BRCAm patients.
- The role that PARPi may have in the treatment of brain metastases of ovarian cancer requires more studies. In the context of radical treatment of brain metastasis (surgery and/or RT), with no evidence of extracranial disease, maintaining treatment with PARPi beyond the brain progression should be considered.
- The evidence of PARPi in brain metastases from OC is primarily based on retrospective studies and case reports. Further prospective studies are needed to identify and validate biomarkers to determine which patients will benefit the most.

## Data availability statement

The raw data supporting the conclusions of this article will be made available by the authors, without undue reservation.

## Ethics statement

Ethical approval was not required for the study involving humans in accordance with the local legislation and institutional requirements. Written informed consent to participate in this study was not required from the participants or the participants’ legal guardians/next of kin in accordance with the national legislation and the institutional requirements. Written informed consent was obtained from the individual(s) for the publication of any potentially identifiable images or data included in this article. Written informed consent was obtained from the participant/patient(s) for the publication of this case report.

## Author contributions

GL: Writing – review & editing, Writing – original draft, Validation, Supervision, Project administration, Methodology, Investigation, Data curation, Conceptualization. TD: Writing – review & editing, Validation, Supervision, Methodology. MI: Writing – review & editing, Validation, Supervision, Methodology, Data curation. LG: Writing – review & editing, Validation, Supervision. EA: Writing – review & editing, Supervision, Validation. AR: Writing – review & editing, Writing – original draft, Validation, Supervision, Methodology, Investigation, Data curation. AS: Writing – review & editing, Writing – original draft, Visualization, Validation, Supervision, Methodology, Investigation, Conceptualization.
